# What is ‘medical necessity’?

**DOI:** 10.1177/14777509231190521

**Published:** 2023-08-01

**Authors:** Dominic JC Wilkinson

**Affiliations:** 1 Oxford Uehiro Centre for Practical Ethics, Faculty of Philosophy, 6396 University of Oxford, UK; 2 John Radcliffe Hospital, Oxford, UK; 3 Murdoch Children's Research Institute, Melbourne, Australia; 4 Centre for Biomedical Ethics, National University of Singapore Yong Loo Lin School of Medicine, Singapore

**Keywords:** Ethics, medical necessity

## Abstract

Imagine that we are considering whether our healthcare system (or insurer) should fund treatment or procedure X. One factor that may be cited is that of so-called ‘medical necessity’. The claim would be that treatment X should be eligible for funding if it is medically necessary, but ineligible if this does not apply. Similarly, (and relevant to the debates in this special issue), if considering whether a particular treatment should be ethically and/or legally permitted, we may wish to distinguish between cases where the treatment is medically necessary, and those were it is not. But what do we mean by this concept? Here I will propose and briefly defend one plausible and practical definition.

Imagine that we are considering whether our healthcare system (or insurer) should fund treatment or procedure X. One factor that may be cited is that of so-called ‘medical necessity’. The claim would be that treatment X should be eligible for funding if it is medically necessary, but ineligible if this does not apply.^1^

Similarly, (and relevant to the debates in this special issue), if considering whether a particular treatment should be ethically and/or legally permitted, we may wish to distinguish between cases where the treatment is medically necessary, and those were it is not.^2^

But what do we mean by this concept? Here I will propose and briefly defend one plausible and practical definition.

**Medical Necessity:** treatment X is ‘medically necessary’ just if, in the absence of X, patient P will suffer from, or has a high chance of suffering from, a significant deterioration in health related wellbeing, or continuation of a significantly lower than normal state of health related wellbeing.

There are two central elements to this concept. The first is related to *need* – here understood as a deviation from normal wellbeing that the patient will experience if they do not receive the intervention. The second is related to the *medical* nature of the need – the wellbeing decrement is related to a state of poor health (it isn’t formally specified in the definition, but we might also think that the treatment or intervention must be ‘medical’ in nature).

This definition has several advantages. It distinguishes medical effects or benefits from medical *need*. It is more stringent than the claim that an intervention is medically ‘appropriate’, or ‘indicated’ or ‘reasonable’. For example, circumcision might be regarded as medically necessary in cases of severe phimosis with recurrent balanitis (narrowing of the foreskin and repeated inflammation/infection) that is unlikely to resolve without surgery. However, circumcision would not be medically necessary in order to reduce the risk of future acquisition of HIV (since this may be prevented in other ways), or cancer of the foreskin (since the risk of this occurring is low).^3^ It also distinguishes cases where an intervention may enhance wellbeing from those where it will alleviate a reduction in wellbeing. For example, an amphetamine prescription may be medically necessary on this definition in a patient with abnormal concentration and attention that is significantly reducing their ability to function at school or the workplace. But it would not be medically necessary (even if it would achieve an identical improvement) in a patient whose baseline concentration and attention were in the normal range.

This definition of medical necessity helps to identify those cases where there is the strongest ethical case for an intervention. It thus may help narrow the range of instances when we wish to fund or permit a particular treatment (if there is a need to restrict funding or permissibility). I do not have space here to explore, but key challenges in applying the concept will include how to define health-related wellbeing and thresholds (e.g. what level or probability of reduced wellbeing would be sufficient) as well as how to deal with cases where other interventions may also be effective but are less desirable.

## Notes

1. This is unlikely to be the only relevant criterion. Other factors will include, for example, how costly the treatment is relevant to its benefit, and how beneficial it is relevant to other alternatives.

2. Once again, medical necessity might be a necessary (pun not intended) but not sufficient condition for legal permissibility.

3. I do not wish to imply here that circumcision would be ‘indicated’ or ‘reasonable’ or ‘appropriate’ for those indications, though some authors have defended that claim.

